# A Newly Developed Synbiotic Yogurt Prevents Diabetes by Improving the Microbiome–Intestine–Pancreas Axis

**DOI:** 10.3390/ijms22041647

**Published:** 2021-02-06

**Authors:** Brandi Miller, Rabina Mainali, Ravinder Nagpal, Hariom Yadav

**Affiliations:** 1 Department of Internal Medicine-Molecular Medicine, Wake Forest School of Medicine, Winston-Salem, NC 27101, USA; bcmiller@wakehealth.edu (B.M.); rnagpal@fsu.edu (R.N.); 2 Department of Pathology-Comparative Medicine, Wake Forest School of Medicine, Winston-Salem, NC 27101, USA; rmainali@wakehealth.edu; 3 Department of Microbiology and Immunology, Wake Forest School of Medicine, Winston-Salem, NC 27101, USA

**Keywords:** diabetes, microbiota, yogurt, milk, dairy, probiotic, prebiotic, synbiotic

## Abstract

The prevalence of type 2 diabetes mellitus (T2D) is increasing worldwide, and there are no long-term preventive strategies to stop this growth. Emerging research shows that perturbations in the gut microbiome significantly contribute to the development of T2D, while microbiome modulators may be beneficial for T2D prevention. However, microbiome modulators that are effective, safe, affordable, and able to be administered daily are not yet available. Based on our previous pro- and prebiotic studies, we developed a novel synbiotic yogurt comprised of human-origin probiotics and plant-based prebiotics and investigated its impact on diet- and streptozotocin-induced T2D in mice. We compared the effects of our synbiotic yogurt to those of a commercially available yogurt (control yogurt). Interestingly, we found that the feeding of the synbiotic yogurt significantly reduced the development of hyperglycemia (diabetes) in response to high-fat diet feeding and streptozotocin compared to milk-fed controls. Surprisingly, the control yogurt exacerbated diabetes progression. Synbiotic yogurt beneficially modulated the gut microbiota composition compared to milk, while the control yogurt negatively modulated it by significantly increasing the abundance of detrimental bacteria such as Proteobacteria and *Enterobacteriaceae*. In addition, the synbiotic yogurt protected pancreatic islet morphology compared to the milk control, while the control yogurt demonstrated worse effects on islets. These results suggest that our newly developed synbiotic yogurt protects against diabetes in mice and can be used as a therapeutic to prevent diabetes progression.

## 1. Introduction

Type 2 diabetes mellitus (T2D) is a chronic metabolic disease that is increasingly prevalent worldwide, affecting more than 10% of the population [1,2]. It is characterized by elevated blood glucose levels (hyperglycemia) due to increased insulin resistance and/or insulin secretion from pancreatic beta (β) cells, which reduces the clearance of blood glucose in peripheral metabolic organs [1]. If not managed well, T2D contributes to the development of several life-threatening comorbidities, including cardiovascular diseases, nephropathy, neuropathy, hyperlipidemia, and organ failure and is also associated with a reduced life expectancy [3,4]. T2D often progresses slowly, with little to no prognosis, and diet is a major risk factor for the development of T2D [5]. Because there are no known long-term strategies to prevent T2D, there is a dire need for the development of therapies that are innovative, cost-effective, safe, and able to be integrated into the diet daily to combat the current T2D epidemic.

Multiple emerging studies indicate that the gut microbiome plays a critical role in the development of T2D and diet is a superlative modulator of the gut microbiome [6,7,8,9]. In particular, T2D is associated with increased Firmicutes and decreased Bacteroidetes (gut dysbiosis) [10,11]. Increased amounts of opportunistic pathogens, such as *Streptococcus* (*S.*) and *Enterobacteriaceae* species, and decreased abundances of beneficial bacteria (probiotics such as *Bifidobacterium* [*B.*] species) and their metabolites, such as short-chain fatty acids (SCFAs), are also prevalent in T2D [10]. Therefore, there is an association between abnormalities in the gut microbiota and T2D, and an abnormal (dysbiotic) microbiota increases the risk of T2D. Thus, therapeutics that are designed to beneficially modulate the gut microbiota may be a feasible approach to prevent T2D. Our previous studies and others have shown that microbiome modulators, such as probiotics and prebiotics, impart beneficial effects on T2D progression in animals and humans [12,13,14,15,16]. We reported that multi-strain probiotics (i.e., VisBiome^®^ (formerly VSL#3), which contains lactobacilli and bacilli strains) [17], yogurt-like products [13,14], a probiotic cocktail containing ten total strains of *Lactobacillus* (*L.*) and *Enterococcus* isolated from the infant gut [18], and prebiotics isolated from sago (palm starch) [12] have antidiabetic effects via modulation of the gut microbiome. However, the inexpensive, safe, and long-term delivery of such regimens for humans—specifically as a preventive strategy—remains challenging. Thus, we have developed a novel synbiotic yogurt that can be delivered safely and inexpensively as a feasible preventive strategy for T2D. The yogurt is a synergistic combination of our newly isolated, well-characterized, and safe human-origin probiotics (five lactobacilli strains) and prebiotics derived from sago. Here, we demonstrate the antidiabetic effects of our newly developed synbiotic yogurt in high-fat diet (HFD)- and streptozotocin (STZ)-induced T2D in mice. We show that the feeding of the synbiotic yogurt significantly improved the gut microbiome composition and intestinal and pancreatic physiology, compared to a commercially available yogurt. Synbiotic yogurt feeding also improved gut permeability by increasing tight junction proteins and reducing inflammation. Our findings highlight the efficacy of human-origin *Lactobacillus* strains in mice and provide evidence that these probiotics, in combination with sago prebiotics, could potentially serve as a therapeutic for T2D in humans.

## 2. Results

### 2.1. Synbiotic Yogurt Protects from Type 2 Diabetes in Mice

**Figure 1:**
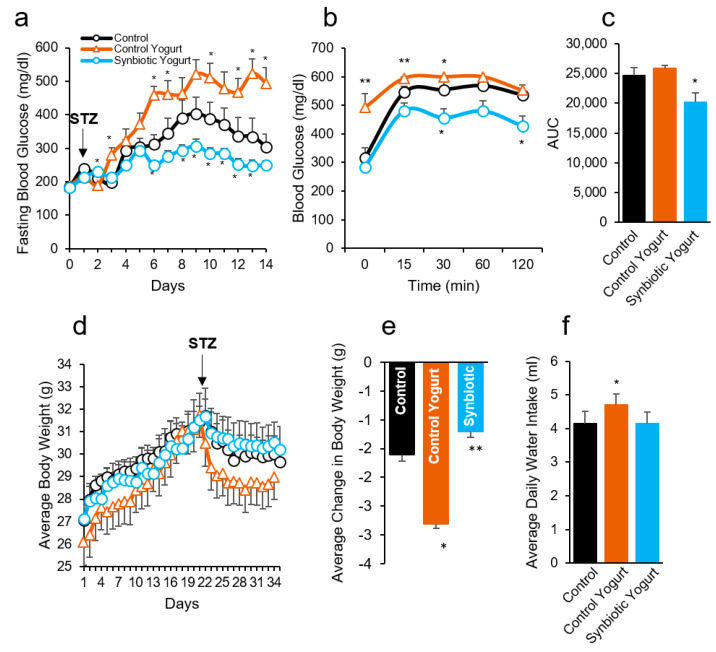
Synbiotic yogurt feeding protects against diabetes progression in mice. (**a**) Synbiotic yogurt feeding significantly reduced the rise in fasting blood glucose levels following STZ administration. (**b**,**c**) Meal tolerance test (**b**) and area under the curve (**c**) were also reduced in synbiotic yogurt-fed mice compared to their controls. (**d**,**e**) Daily body weight (**d**) and average change in body weight following STZ administration (**e**). (**f**) Average daily water intake. Values are mean ± standard error of the mean (error bars). Values with * *p* < 0.05; ** *p* < 0.001 are statistically significant. AUC, Area under curve; STZ, Streptozotocin.

Herein, we developed a sensory evaluated and firm low-fat (2% milk fat) synbiotic yogurt. To determine the effects of this newly developed synbiotic yogurt on the progression of HFD-/STZ-induced T2D, we fed mice a HFD supplemented with 15% (*w*/*v*) synbiotic yogurt and compared the phenotypes of these mice to the following two control groups: (1) HFD supplemented with 15% (*w*/*v*) boiled 2% milk (control) and (2) HFD supplemented with 15% (*w*/*v*) of a commercially available yogurt (control yogurt), which was selected based on availability, fat content, and flavor. The synbiotic yogurt-fed mice demonstrated significantly lower fasting blood glucose (FBG) levels, while the FBG levels in the milk- and control yogurt-fed groups increased (Figure 1a). Surprisingly, control yogurt-fed mice exhibited a significantly higher rise in FBG levels than the milk control mice. Furthermore, the blood glucose rise during the meal tolerance test (MTT) was significantly lower in the synbiotic yogurt-fed mice, compared to the control groups; again, these values were notably higher in the control yogurt-fed mice, compared to the other groups (Figure 1b). Similar trends were also observed in the area under the curve during MTT (Figure 1c). Insulin sensitivity measured by insulin tolerance was not significantly different among the groups; however, the blood glucose levels and area under the curve during the test were lower in the synbiotic yogurt-fed mice compared to both controls (Supplementary Figure S1a,b). These results indicate that the synbiotic yogurt protects against HFD-/STZ-induced T2D by maintaining lower FBG levels and preserving meal tolerance without impacting insulin sensitivity.

STZ is known to reduce body weight while causing severe hyperglycemia/T2D [19,20]. Interestingly, synbiotic yogurt feeding suppressed the weight-reducing effects of STZ, while the control yogurt-fed mice lost the most weight during the two weeks following STZ administration (Figure 1d,e). This suggests that the synbiotic yogurt reduced the severity of low-dose STZ-induced T2D in mice, while the control yogurt enhanced it. Although food intake was similar among the groups (Supplementary Figure S1c), the water intake was significantly higher in the control yogurt-fed mice, compared to the milk- and synbiotic yogurt-fed groups (Figure 1f). This suggests that polydipsia (excessive thirst) and polyuria (excessive urination), which are common features of diabetes, were prevalent in the control yogurt-fed mice. Overall, these results indicate that synbiotic yogurt feeding protected from T2D progression in mice, while the control yogurt aggravated it. However, the mechanisms contributing to these trends are unclear. 

### 2.2. Synbiotic Yogurt Protects Against Streptozotocin-Induced Pancreatic Islet Damage

**Figure 2:**
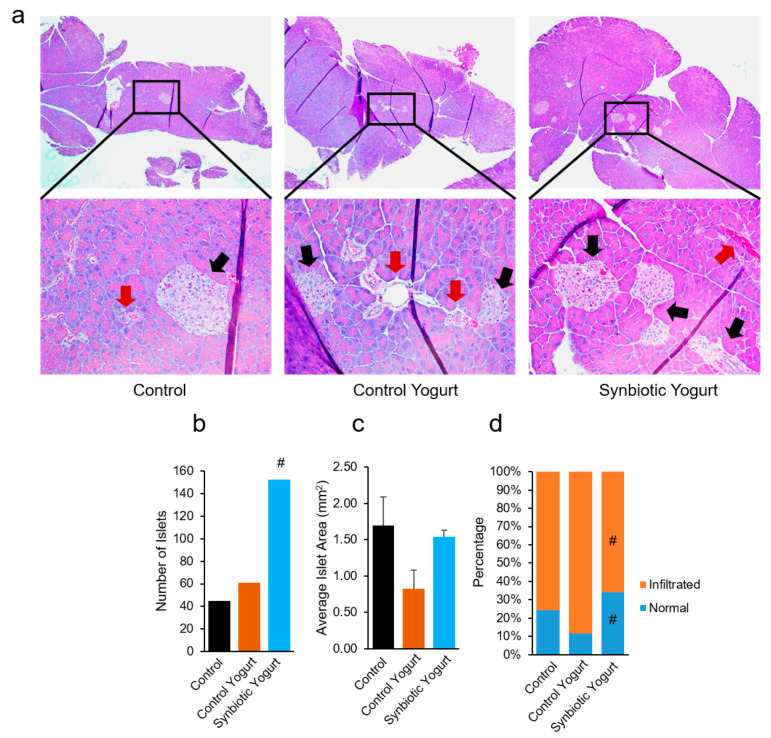
Synbiotic yogurt feeding preserves pancreatic islets and protects against streptozotocin-induced islet damage. (**a**) Histological analyses of pancreatic sections. Black arrows point to islets with normal morphology, while red arrows point to those classified as infiltrated or involuted in shape. (**b**) The total number of islets counted from pancreas sections in each group. (**c**) Average islet area. (**d**) Percent of infiltrated and normal islets in each group. Values are mean ± standard error of the mean (error bars). Values with ^#^ *p* < 0.05 are statistically significant compared to the control yogurt group.

To evaluate the impacts of synbiotic yogurt feeding on STZ-induced damage of pancreatic islets, we counted the number of preserved islets, calculated the average islet area, and determined the proportion of normal islets versus infiltrated (inflamed) islets. Interestingly, the number of islets was significantly higher in the synbiotic yogurt-fed mice compared to the milk- and control yogurt-fed groups (Figure 2a,b). Although the differences were not statistically significant, the average islet area in the control yogurt-fed mice was lower than the areas observed in the milk- and synbiotic yogurt-fed mice (Figure 2c). We also found that there were significantly more normal islets and less inflamed islets in the synbiotic yogurt group, compared to the control yogurt group (Figure 2d). Normal islets were classified as those that had no obvious infiltration or signs of inflammation, while we classified infiltrated islets as those that had obvious immune cell infiltration and were involuted in shape. Overall, these results demonstrated that the feeding of synbiotic yogurt protected against pancreatic islet damage induced by HFD and STZ, which reduced the progression of T2D in mice. 

### 2.3. Synbiotic Yogurt Beneficially Modulates the Gut Microbiome in Mice with Type 2 Diabetes

**Figure 3:**
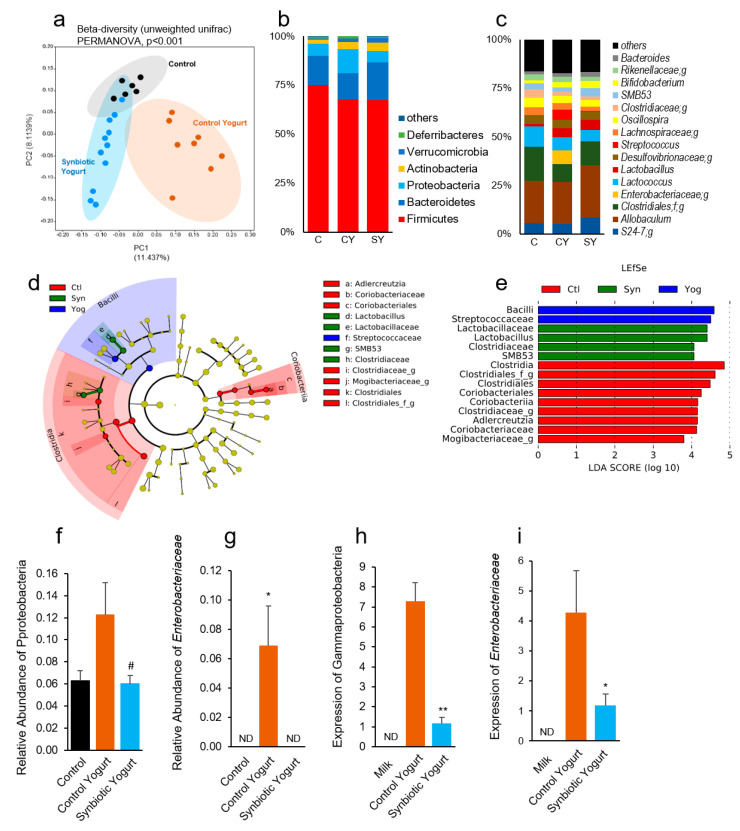
Synbiotic yogurt feeding protects against high-fat diet-induced gut microbiome dysbiosis. (**a**) Principal component analysis showing β-diversity clustering of the gut microbiome of mice fed a high-fat diet supplemented with milk, control yogurt, and our synbiotic yogurt. (**b**) Major bacterial phyla. (**c**) Major bacterial genera. (**d**,**e**) Cladogram (**d**) and linear discrimination analysis effect size (LefSe) (**e**) showing clustering in the gut microbiome. (**f**,**g**) Abundances of Proteobacteria (**f**) and *Enterobacteriaceae* (**g**) in mouse feces. (**h**,**i**) Expression of Gammaproteobacteria (**h**) and *Enterobacteriaceae* (**i**) in milk, control yogurt, and our synbiotic yogurt using real-time PCR. Control yogurt was used as the control because these bacteria were not detected in boiled milk. Values are mean ± standard error of the mean (error bars). * *p* < 0.05; ** *p* < 0.001; ^#^ *p* < 0.05 compared to control yogurt. ND, Not detected; SY/Syn, Synbiotic yogurt; CY/Yog, Control yogurt.

The gut microbiota has a critical role in T2D pathology and synbiotics have emerged as superlative modulators of the gut microbiota [15,21]. We found that the β-diversity of the gut microbiome was significantly different among the groups (PERMANOVA *p* < 0.001; Figure 3a), indicating that the milk and yogurt diets significantly changed microbial diversity in the gut of mice with T2D. However, we did not observe significant differences in α-diversity indices, such as the observed OTUs (operational taxonomic units), Shannon index, and Chao1 (Supplementary Figure S2a,b,c). The relative abundances of bacterial phyla and genera were different among the groups (Figure 3b,c). In particular, the abundance of Firmicutes was lower in the synbiotic and control yogurt groups, while the abundance of Bacteroidetes was higher in the synbiotic yogurt-fed group, with no statistically significant differences (Supplementary Figure S2d,e). Thus, the ratio of Firmicutes to Bacteriodetes was slightly higher in the synbiotic yogurt-fed group compared to both controls, with no significant differences (Supplementary Figure S2f). In addition, the abundances of *Lactobacillus* and *Bifidobacterium* were higher in both yogurt groups compared to the milk control group (Supplementary Figure S2g,h). *Streptococcus* was significantly more abundant in the control yogurt group, compared to the control and synbiotic yogurt groups (Supplementary Figure S2i). This could be attributed to the *S. thermophilus* culture present in the control yogurt. The linear discriminant analysis (LDA) effect size (LefSe) cladogram and LDA score also show restored *Lactobacillus* in the synbiotic yogurt group, increased *Streptococcaceae* in the control yogurt group, and increased *Clostridiales* and *Clostridiaceae* in the milk control group (Figure 3d,e).

In addition, we found that the control yogurt significantly increased the abundances of Proteobacteria and *Enterobacteriaceae*, which are primarily comprised of enteropathogens and Gram-negative species, compared to the milk control and synbiotic yogurt groups (Figure 3f,g). We further investigated the potential reasons for such changes and our real-time PCR-based quantification confirmed that the abundances of *Enterobacteriaceae* (measured by the *rpoB* gene) and Proteobacteria (measured by the *Gamma* gene) were significantly higher in the feces of T2D mice fed the control yogurt. While determining if either yogurt was the source of these bacteria, we found that the relative abundances of Gammaproteobacteria and *Enterobacteriacae* were also significantly higher in the control yogurt compared to the milk, where both were not detected, and the synbiotic yogurt (Figure 3h,i). As expected, the abundances of fecal Proteobacteria and *Enterobacteriacae* were positively correlated with FBG levels in the control yogurt group, but not the other groups (data not shown). These results suggest that the control yogurt was the source of Proteobacteria and *Enterobacteriacae* and likely contributed to the colonization of enteropathogens in the gut of control yogurt-fed mice and the increased progression of diabetes in these mice.

### 2.4. Synbiotic Yogurt Improves Gut Barriers and Reduces Inflammation in the Gut–Liver–Pancreas Axis

**Figure 4:**
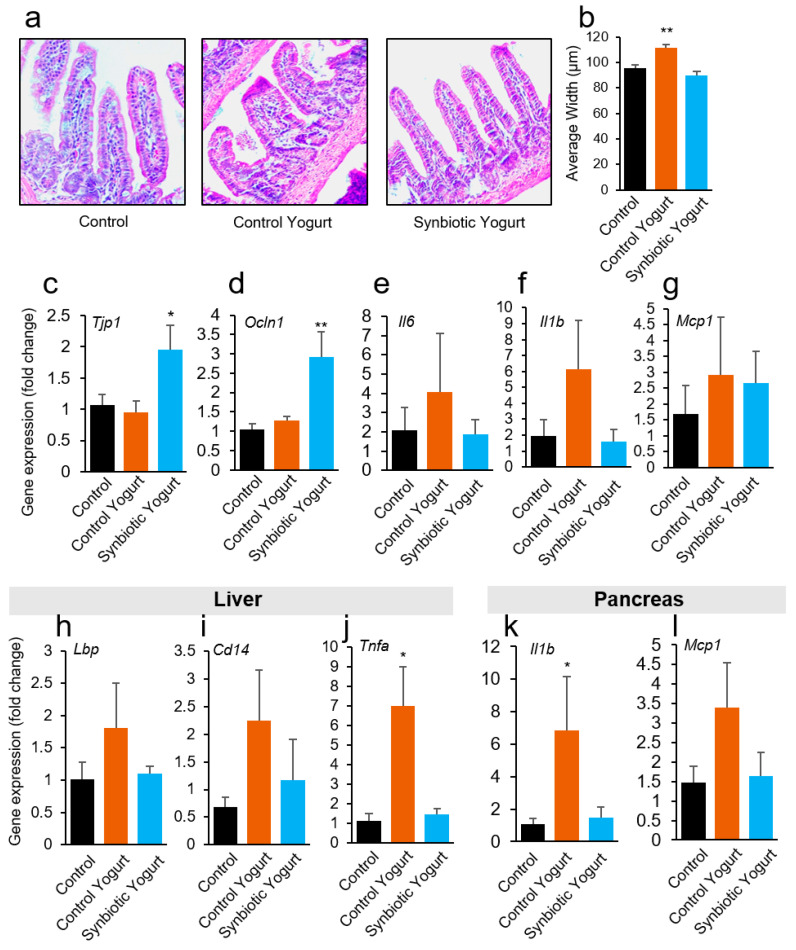
Synbiotic yogurt feeding reduces leaky gut and gut inflammation in the gut–liver–pancreas axis. (**a**,**b**) Synbiotic yogurt feeding protects against intestinal (ileum) villi damage (**a**), which is characterized by decreased villi width and less inflamed villi (**b**). (**c**,**d**) Synbiotic yogurt feeding increases the expression of tight junction proteins Tjp-1 (**c**) and Ocln1 (**d**) in the ileum compared to the control groups. (**e**–**g**) Control yogurt feeding worsened the expression of inflammatory markers such as IL-6 (**e**), IL-1β (**f**), and MCP-1 (**g**) in the ileum, in comparison to milk-fed mice, while synbiotic yogurt feeding reduced it. (**h**–**l**) In addition, control yogurt feeding increased the expression of leaky gut markers LBP (**h**) and CD14 (**i**) and inflammatory marker TNF-α (**j**) in the liver, while synbiotic yogurt lowered their levels close to the control. Similarly, the control yogurt increased the expression of IL-1β (**k**) and MCP-1 (**l**) in the pancreas, while the synbiotic yogurt lowered them. Values are mean ± standard error of the mean (error bars). Values with * *p* < 0.05; ** *p* < 0.001 are statistically significant. CD14, Cluster of differentiation 14; IL, Interleukin; LBP, Lipopolysaccharide binding protein; MCP-1, Monocyte chemoattractant protein-1; Ocln1, Occludin-1; Tjp-1, Tight junction protein-1; TNF-α, Tumor necrosis factor-α.

Furthermore, we determined the impacts of our yogurt on intestinal (ileum) morphology. Although there were no statistically significant differences in the villi length (data not shown), we observed that the villi width was larger in the control yogurt-fed mice compared to the milk- and synbiotic yogurt-fed groups (Figure 4a,b). Because wider villi may be a sign of swelling or inflammation [22], our results suggest that the intestine of mice fed the control yogurt may have had higher levels of inflammation. Interestingly, the expression of barrier function proteins, such as tight junction protein-1 (Tjp-1)/Zonulin-1 and occludin-1 (Ocln1), were significantly higher in the ileum of synbiotic-yogurt fed mice compared to the milk- and control yogurt-fed groups (Figure 4c,d). This suggests that synbiotic yogurt feeding improved gut barrier functions to preserve gut permeability and prevent leaky gut. Furthermore, the expression of pro-inflammatory markers, such as interleukin-6 (IL-6), IL-1β, and monocyte chemoattractant protein-1 (MCP-1) were reduced in intestinal tissues (ileum) of T2D mice fed synbiotic yogurt, compared to both control groups (Figure 4e–g). Although there were no statistically significant differences, which was partly due to the high variation in the expression from mouse-to-mouse, the observed trends suggest that synbiotic yogurt feeding reduced inflammation in the intestinal tissues. Intestinal leakiness and inflammatory signals can pass through the liver and migrate to the pancreas through portal circulation [23]. Interestingly, we observed increases in leaky gut markers, such as lipopolysaccharide binding protein (LBP) and cluster of differentiation 14 (CD14) antigen, as well as increases in pro-inflammatory markers, such as tumor necrosis factor-alpha (TNF-α), in the liver of control yogurt-fed mice compared to the milk- and synbiotic yogurt-fed groups (Figure 4h–j). Additionally, IL-1β and MCP-1 levels were higher in the pancreas of control yogurt-fed T2D mice in comparison to the milk- and synbiotic yogurt-fed groups (Figure 4k,l). These results suggest that the control yogurt promoted leaky gut and the upregulation of inflammatory signals in the intestine, which migrated to the liver and pancreas, while synbiotic yogurt reversed them by increasing the expression of tight junction proteins.

## 3. Discussion

The prevalence of T2D is increasing and the availability of feasible, safe, and long-term preventive and therapeutic strategies is limited. Emerging evidence shows that the gut microbiome plays a key role in the pathology of T2D and is a therapeutic target for T2D due to its high plasticity [7,9,24]. Here, we developed a novel synbiotic yogurt comprised of five strains of human-origin probiotics (lactobacilli) [18] and newly-isolated prebiotics from sago starch [12], which have both demonstrated strong preventive effects against metabolic disease and T2D in vivo. We showed that feeding synbiotic yogurt significantly reduced hyperglycemia in mice, compared to a milk-fed control group. Interestingly, we also showed that supplementing the HFD with a commercial yogurt, which was selected based on fat content and flavor, significantly increased hyperglycemia in HFD-/STZ-induced T2D mice. These results indicate that our novel synbiotic yogurt reduced the progression of T2D upon HFD and STZ administration, while the control yogurt exacerbated it. Our findings for the control yogurt were surprising because yogurt consumption (especially low-fat probiotic yogurt) has been shown to suppress hyperglycemia and other markers associated with T2D progression [25,26]. The detection of Proteobacteria and *Enterobacteriaceae* in the gut of control yogurt-fed mice was also striking and led us to conduct additional quantitative PCR analyses to determine the possible mechanisms contributing to this colonization. Based on our results, we speculate that the control yogurt was the source of these pathogenic bacteria and, therefore, exacerbated diabetes progression via increased inflammation and gut permeability; however, the precise mechanisms underlying rapid diabetes progression in the control yogurt-fed mice are not known. We repeated our experiments in three different batches with different lots of yogurt from the store and found similar results in all of the tested batches. Therefore, the effects of the control yogurt were not due to lot-to-lot variabilities. Our findings raise concerns regarding the effects of other commercial yogurts on the incidence and progression of T2D. Thus, our future studies may address the efficacy and safety concerns of different commercial yogurts on the progression of T2D.

Polydipsia (excessive thirst) is a common characteristic of diabetes and is associated with polyuria (increased urination) [27]. Interestingly, control yogurt-fed mice demonstrated a significantly higher water intake, further suggesting that the control yogurt promoted diabetes in mice. Conversely, the synbiotic yogurt showed no effects on water intake, suggesting that it preserved normal water intake and did not aggravate T2D progression. A HFD and a single low dose of STZ create stress in pancreatic β cells, which leads to insulin deficiency; over time, insulin resistance develops due to fat accumulation in peripheral organs that uptake glucose (i.e., liver) and the death of β cells in the pancreas [28,29]. Interestingly, synbiotic yogurt feeding preserved islets and their morphology, compared to milk. On the other hand, the control yogurt group exhibited a similar number of islets compared to the control group; however, the islet area was lower than that in the milk control and synbiotic yogurt groups, suggesting that the control yogurt may have promoted more β-cell death, leading to the development of smaller islet patches within the pancreas. Furthermore, the number of highly infiltrated/involuted islets was significantly higher in the control yogurt-fed group, compared to the milk- and synbiotic yogurt-fed mice. Pancreas sections with less islets, smaller islets, and more infiltrated/involuted islets are likely to exhibit increased β cell death [30,31] and involuted islet structures often occur due to increased inflammation [32]. Our data suggest that the control yogurt-fed mice exhibited an increased number of inflamed islets in the pancreas, while the synbiotic yogurt-fed mice had less, compared to the milk-fed control mice. The synbiotic yogurt also preserved healthy villus structures in the intestine, compared to the control yogurt. Supplementation of probiotics, such as species of *Lactococcus, Lactobacillus, Bifidobacterium*, and *Streptococcus*, as well as synbiotics, preserves healthy villus architecture, including the height, width, and surface area of the villi [33,34,35]. Our findings suggest that the synbiotic yogurt may have protected the villi from damage, which is important for maintaining gut integrity and reducing inflammation. On the other hand, the control yogurt significantly increased the villi width, which is a sign of increased inflammation and may promote damage to the intestinal epithelium. 

The gut microbiome plays a crucial role in the development of diabetes and our results demonstrated that the synbiotic yogurt beneficially modulated the microbiome composition, while the control yogurt promoted the growth of detrimental bacteria in the gut of diabetic mice. One potential mechanism by which the gut microbiome influences metabolic disease and T2D is via the fermentation of indigestible fibers into SCFAs (i.e., acetate, propionate, and butyrate) and others, and these metabolites can regulate blood glucose levels and insulin release [36]. Supplementation of other multi-strain probiotics, which include species of *Bifidobacterium*, *Lactobacillus*, and *Streptococcus* have demonstrated beneficial modulations in the gut microbiome, which are associated with increased secretion of SCFAs (mainly butyrate), improved glycemic control and insulin sensitivity, and reduced gut permeability in T2D [17,37,38,39]. Therefore, the inclusion of our newly-isolated human-origin *Lactobacillus* probiotics in our synbiotic yogurt may have prevented T2D progression by beneficially shifting the gut microbiome composition (i.e., increasing bifidobacteria and preventing the colonization of enteropathogens), leading to the production of beneficial metabolites and improved gut physiology. Analyzing the SCFAs and other metabolites in the gut and portal circulation could be of interest in future studies to further explain the mechanisms by which the gut microbiome signature influenced the progression of T2D in the synbiotic yogurt-fed mice.

Furthermore, one of the most notable findings was that the control yogurt significantly increased the growth of pathogenic, Gram-negative commensal bacteria such as Proteobacteria and *Enterobacteriaceae*. We also found that the control yogurt itself had a higher content of *Enterobacteriaceae*, suggesting why these bacteria were enriched in the gut of control yogurt-fed T2D mice. An increased abundance of Gram-negative bacteria is associated with an increased incidence of diabetes [40,41]. This might be because the Gram-negative bacterial cell wall is a rich source of lipopolysaccharide (LPS), which is highly inflammatory [42]. Therefore, increases in Gram-negative bacteria/*Enterobacteriaceae* heightens the burden of LPS in the gut, which may lead to increased gut permeability (leaky gut), inflammation, and endotoxemia [41,42]. Interestingly, our results demonstrated that the control yogurt significantly decreased the expression of tight junction proteins (Tjp-1 and Ocln1) and increased inflammatory markers in the gut, which negatively impacted the intestinal barrier function and increased inflammation in the gut. However, these effects were not limited to the gut as we observed significant increases in leaky gut and inflammatory markers, such as LBP and CD14, in the liver, as well as inflammation in the pancreas. These results indicate that increased abundances of detrimental bacteria in the gut induce abnormalities in gut barriers, leading to leaky gut and the non-specific diffusion of pro-inflammatory molecules, such as LPS, from the gut to portal vein circulation. After reaching liver circulation, LPS binds LBP to neutralize LPS and induce inflammation [43,44]. The circulation of LPS and LBP and inflammation also extends to the pancreas, which exacerbates the production of pro-inflammatory cytokines and leads to inflammation of the gut–liver–pancreas axis. We observed this in our results, which demonstrated that control yogurt feeding increased inflammation in the gut–liver–pancreas axis and promoted the growth of Gram-negative bacteria in the gut. These associations may underlie the mechanisms by which the control yogurt promoted diabetes progression in mice in our study. On the other hand, the synbiotic yogurt suppressed leaky gut and inflammation in the gut–liver–pancreas axis, suggesting that controlling leaky gut and inflammation in this axis may be a successful preventive and therapeutic approach for diabetes. Several gut microbiota modulators, such as probiotics, prebiotics, and dietary interventions have been investigated; however, their effects remain variable and the adherence to and implementation of these modalities is challenging. Our newly developed synbiotic yogurt may be a stable food supplement that will be easy to administer daily to patients with T2D or individuals at a high risk of developing T2D.

## 4. Materials and Methods

### 4.1. Culturing Probiotic Bacteria

Five human-origin strains of *Lactobacillus* probiotics (*L. plantarum* D13-4, *L. rhamnosus* D7-5, *L. paracasei* D3-5, *L. plantarum* D6-2, and *L. rhamnosus* D4-4) were isolated on De Man, Rogosa, Sharpe (MRS) agar, as previously described, and transferred to a liquid medium (MRS) [18]. To prepare stocks, we inoculated 1 mL of bacterial suspension into 45 mL of MRS and grew the cultures for 12–24 h at 37 °C. Stocks were kept at 4 °C for up to two months. Isolates were maintained in glycerol at −80 °C for long-term preservation.

### 4.2. Isolation and Small-Scale Production of Sago Prebiotics

Sago fibers were prepared using our previously established protocol and modified methods for preparing resistant starches [12,45]. Briefly, we suspended sago seeds (Jalipur Millers, UK) in dH2O at a 1:20 dilution (*w*/*v*) and boiled the suspension on a hotplate for ten minutes with a magnetic stirrer. The suspension was autoclaved at 121 °C and 1.2 atm for one hour and kept at 4 °C overnight to allow retrogradation. The retrograded starch was hydrolyzed by adding 2 U/mL of α-amylase (Sigma–Aldrich, St. Louis, MO, USA) and 1 mL/L of Pullulanase microbial (Sigma–Aldrich, St. Louis, MO, USA) and was incubated for 24 h in a shaker incubator (150 rpm at 70 °C). The suspension was centrifuged at room temperature (25 °C) for ten minutes at 10,000 rpm. The supernatant was decanted and pellets were resuspended in 1 L of dH2O, and the enzymatic hydrolysis and centrifugation processes were repeated. The obtained pellets were freeze-dried (LABCONE, Freezone 4.5, Kansas, MO, USA) after overnight storage at −80 °C.

### 4.3. Development of Synbiotic Yogurt

Based on our previous studies, we developed a synbiotic yogurt that was firm, sensory acceptable to humans, and comprised of sufficient pro- and prebiotics [12,18]. After evaluating the coagulation and pH profiles of the individual strains, five lactobacilli, five enterococci, and the ten-strain cocktail in skim, 2%, and whole milk, we found that the five lactobacilli strains were able to form curd in the 2% fat and whole milks only. Enterococci strains did not make sufficient curd and worsened the coagulation and yogurt texture when added with the lactobacilli; therefore, they were not used in the final product. Although the quality of the strains in whole milk was comparable to that of 2% milk, we omitted whole milk because it has a higher fat content, which may not be ideal for people who are obese or have diabetes. Despite the formation of curd with the five lactobacilli strains and 5% (*w*/*v*) sago, the syneresis rate (percent whey release) was high and the yogurt firmness was low. Therefore, we added pectin to the yogurt in order to develop a sensory acceptable and highly favorable yogurt. Using a pectin gradient, we found that a concentration of 0.175% pectin (*w*/*v*) improved the firmness, reduced the syneresis rate, and increased sensory evaluation scores the most. We used 5% sago (*w*/*v*) based on our previous study, in which we optimized the amount of sago in order to achieve a biologically functional dose of prebiotics in vivo [12]. 

### 4.4. Preparation of Synbiotic Yogurt

We prepared our yogurt by inoculating 200 µL of each bacterial stock individually into 10 mL of MRS and growing it for 18–24 h at 37 °C. Optical density (OD) values (600 nm) were used to calculate the volume of bacterial suspension to be inoculated into the milk so that 1 mL of milk contained 10^8^ colony-forming units of each strain. Bacterial pellets were washed and centrifuged three times with 45 mL of 0.9% sodium chloride before being suspended into 100 mL of boiled low-fat milk (2% fat; Great Value, Austin, TX, USA), which was supplemented with 5% and 0.175% (*w*/*v*) of sago and pectin, respectively. After thorough mixing, the milk suspension was pipetted into 10 mL aliquots and fermented for 14–16 h at 37 °C, or until the pH dropped between 3.7 and 4.5. Fresh yogurt was prepared every seven days.

### 4.5. Selection of a Commercially-Available Yogurt Control

To compare the effects of our synbiotic yogurt to a commercially-available yogurt, we selected a yogurt which (1) was available at multiple grocery stores, (2) was prepared with 2% milk, and (3) did not include added sugars or flavors. We selected a Greek Yogurt, which contains five live probiotic cultures (*L. bulgaricus*, *S. thermophilus*, *L. acidophilus*, *Bifidus*, and *L. casei*). A fresh container of yogurt was purchased every seven days.

### 4.6. Animal Studies

#### 4.6.1. Mice

Twenty-five male C57BL/6J mice (age: 10-12 weeks) were randomized into three groups, which were fed the following diets: (1) Control (n = 6): HFD (60% fat; Research Diets Inc., New Brunswick, NJ, USA) supplemented with 15% (*w*/*v*) boiled 2% milk (Great Value, Austin, TX, USA), (2) Control Yogurt (n = 8): HFD with 15% (*w*/*v*) of the selected commercial yogurt, and (3) Synbiotic Yogurt (n = 11): HFD supplemented with 15% (*w*/*v*) synbiotic yogurt. Diet and water were provided ad libitum; diet was replaced daily, while water was replaced weekly. Mice studies were designed as five-week longitudinal studies. All animal studies and procedures were approved by the Animal Research Program’s Institutional Animal Care and Use Committee of the Wake Forest School of Medicine. 

#### 4.6.2. Development of Type 2 Diabetes Mellitus in Mice

We induced T2D in mice through HFD feeding and a single intraperitoneal dose (90 mg/kg body weight) of STZ. STZ is specifically toxic to pancreatic β cells and leads to β-cell necrosis/partial cell death via its glucose transporter-mediated accumulation, DNA damage, and the production of reactive oxygen species and/or nitric oxide [46,47]. STZ was administered after three weeks of HFD, according to a previous study, following a six-hour fast [48].

#### 4.6.3. Daily Measurements

Measurements of body weight, water intake, and food intake were recorded at the same time daily. Starting on the day of STZ administration, FBG was measured daily after a six-hour fast via tail bleed using the EvenCare ProView Blood Glucose Monitoring System (Medline Industries, Inc., Mundelein, IL, USA) to monitor diabetes progression.

#### 4.6.4. Meal Tolerance Tests

MTTs were performed twice at baseline and once at endpoint. All mice received two baseline MTTs (one with milk and one with milk or either yogurt, depending on the group). The meal contained 570 mg of Vanilla Ensure Original Nutrition Powder (Abbott Laboratories, Columbus OH, USA) and 30 mg of dextrose, which were dissolved in 1.8 mL of liquid and vortexed thoroughly. For baseline assays, 0.9 mL of dH2O and 0.9 mL of milk/yogurt (depending on the group) were used, while 1.8 mL of dH2O was used at endpoint. The meal (200 µL per mouse) was administered via oral gavage, following an eight-hour fast. Blood glucose measurements were recorded before oral gavage, and 15, 30, 60, and 120 min after, via tail bleed, using the EvenCare ProView Blood Glucose Monitoring System. 

#### 4.6.5. Insulin Tolerance Tests

An insulin tolerance test was performed at baseline and endpoint, following a six-hour fast. Insulin (Humulin R; Lilly USA, LLC, Indianapolis, IN, USA) was administered intraperitoneally at a dose of 0.5 U/kg body weight. Blood glucose measurements were recorded before injection and 15, 30, 60, and 120 min after, via tail bleed, using the EvenCare ProView Blood Glucose Monitoring System. 

### 4.7. Fecal Microbiome Analysis

Fresh feces were collected at endpoint in a sterile 1.5 mL Eppendorf tube and were immediately frozen at −80 °C until use. 16S rRNA gene amplification and sequencing were performed using our previously established methods [12,18,49,50,51,52,53]. Briefly, genomic DNA was extracted from ~100 g of solid feces using the Qiagen QIAamp PowerFecal Pro DNA Kit (Qiagen Inc., Valencia, CA, USA), according to the manufacturer’s instructions. The 515F (barcoded) and 806R primers were used, in accordance with the Earth Microbiome Project protocol [54], to amplify the V4 region of the 16S bacterial rRNA gene, with minor modifications [55]. AMPure^®^ magnetic amplification beads (Agencourt) were used to purify the amplicons, which were quantified using the Qubit-3 fluorimeter (InVitrogen). Purified PCR products were pooled; this resulting pool was quantified, normalized (4 mm), denatured and diluted (8 pM), and sequenced using the Illumina MiSeq sequencer and the Miseq reagent kit v3. The Quantitative Insights into Microbial Ecology software was used to de-multiplex, quality filter, cluster, and analyze the sequences. LDA and cladograms were constructed at the genus level using LefSe on the Galaxy platform (https://huttenhower.sph.harvard.edu/galaxy/ (accessed on 1 February 2021)) [56]. For the principal component analysis (PCA), each dot represents the feces from one mouse. 

### 4.8. Yogurt Pathogen Analysis

Further analysis was conducted to determine the source of pathogenic bacteria (Proteobacteria and *Enterobacteriaceae*), which were observed in the fecal microbiome analysis. Genomic DNA was extracted from mouse feces, milk, and both yogurts using the Qiagen QIAamp PowerFecal DNA Kit (Qiagen, Valencia, CA, USA), according to the manufacturer’s instructions. The target gene for *Enterobacteriaceae* was *rpoB* (primers: F: 5′-CAGGTCGTCACGGTAACAAG-3′; R: 5′-GTGGTTCAGTTTCAGCATGTAC-3′), while the target gene for Proteobacteria (specifically Gammaproteobacteria) was *Gamma* (primers: F: 5′- CMATGCCGCGTGTGTGAA-3′; R: 5′- ACTCCCCAGGCGGTCDACTTA-3′). Quantitative gene expression was performed using real-time PCR (ABI 7500; Applied Biosystems). The 16S rRNA gene served as the internal control and all samples were run in triplicate. Because these genes were not detected in the milk, results are reported as the fold change in gene expression in comparison to the control yogurt (for the yogurts) and control mouse feces (for feces).

### 4.9. Histological Analyses

Ileum and pancreas tissues were harvested and fixed in 10% phosphate-buffered formalin for 24 h, followed by 70% ethanol, and were stored at 4 °C until use. Tissues were processed into paraffin blocks, sectioned at 6 µm, and stained with hematoxylin and eosin. One hundred villi were counted from representative ileum sections in each group and their length and width were measured at 20x magnification. For pancreas analyses, the total number of islets, the average islet area, and general morphology (normal versus infiltrated) were evaluated using four pancreas sections per group. Photos of pancreas sections were captured at 4× magnification. A light microscope was used to observe stained tissue sections and images were captured using a 9-MP digital camera (MU900, AmScope). All image analyses were conducted using ImageJ software.

### 4.10. Gene Expression

Ileum, liver, and pancreas tissues were harvested, snap-frozen in liquid nitrogen, and stored at −80 °C until use. Total RNA was isolated from approximately 30 mg of each tissue for six randomly selected mice in each group using the Qiagen RNeasy Kit (Qiagen, Gaithersburg, MD, USA) after thorough homogenization and sonication. RNA was reverse transcribed to complementary DNA using the ABI reverse transcription kit, as previously described [12,17,51,52,57]. We used real-time PCR to quantify the expression of inflammatory markers (IL-6, IL-1β, TNF-α, and MCP-1) and gut permeability markers (Tjp1, Ocln-1, LBP, and CD14) in these tissues (ABI 7500; Applied Biosystems). Gene expression was recorded as the fold change, in comparison to the 18S internal control, as previously described [12,17,51,52].

### 4.11. Statistical Analyses

All data are expressed as mean ± standard error of the mean, unless otherwise noted. ImageJ software was used for histological analyses. PERMANOVA was used to compare β-diversity measures and analyze the inter- and intra-group distances on the PCA in the fecal microbiome analysis. The Chi-squared test of homogeneity was used to compare pancreatic islet morphology. All other comparisons between groups were made using the t-test or ANOVA. For all analyses, *p* < 0.05 was considered statistically significant. 

## 5. Conclusions

Our studies demonstrated the efficacy of a newly developed synbiotic yogurt containing five strains of human-origin probiotic lactobacilli and newly-isolated prebiotics from sago starch on the development of T2D in mice. Synbiotic yogurt feeding was associated with beneficial modulation of the gut microbiome and reduced leaky gut and inflammation in the gut-liver-pancreas axis. This newly developed synbiotic yogurt may be an alternative and/or staple food to prevent or treat diabetes.

## Data Availability

Data will be available upon reasonable request to the study team, following Wake Forest School of Medicine guidelines.

## Supplementary Materials

The following are available online at https://www.mdpi.com/1422-0067/22/4/1647/s1, Figure S1. Synbiotic yogurt feeding does not affect insulin sensitivity or food intake. Figure S2. Control and synbiotic yogurt feeding does not significantly affect α-diversity measures but modulates the gut microbiome at the phyla and species levels.
