# Supplementary material for: A Newly Developed Synbiotic Yogurt Prevents Diabetes by Improving the Microbiome–Intestine–Pancreas Axis
Source: Int J Mol Sci. 2021 Feb 6;22(4):1647. doi: 10.3390/ijms22041647 (PMC7915949; doi:10.3390/ijms22041647)
Supplement: Supplementary file 1 [file ijms-22-01647-s001.pdf]

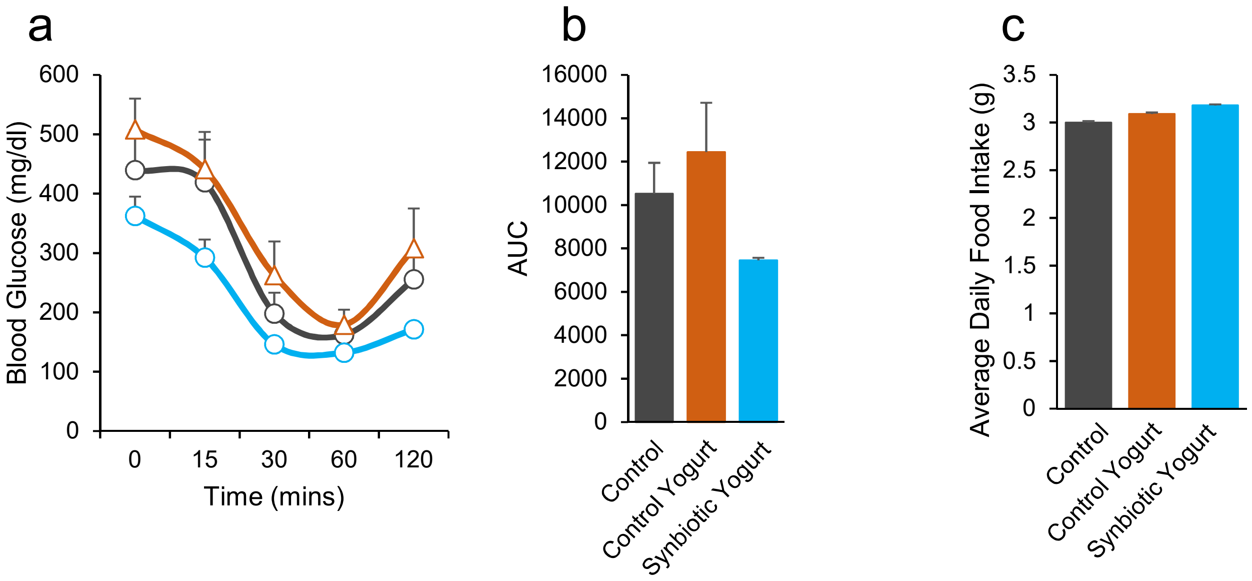

Supplementary Figure S1. Synbiotic yogurt feeding does not affect insulin sensitivity or food intake. (a,b) Insulin sensitivity during the insulin tolerance test (a) and area under the curve analysis (b). (c) Average daily food intake.

AUC, Area under the curve

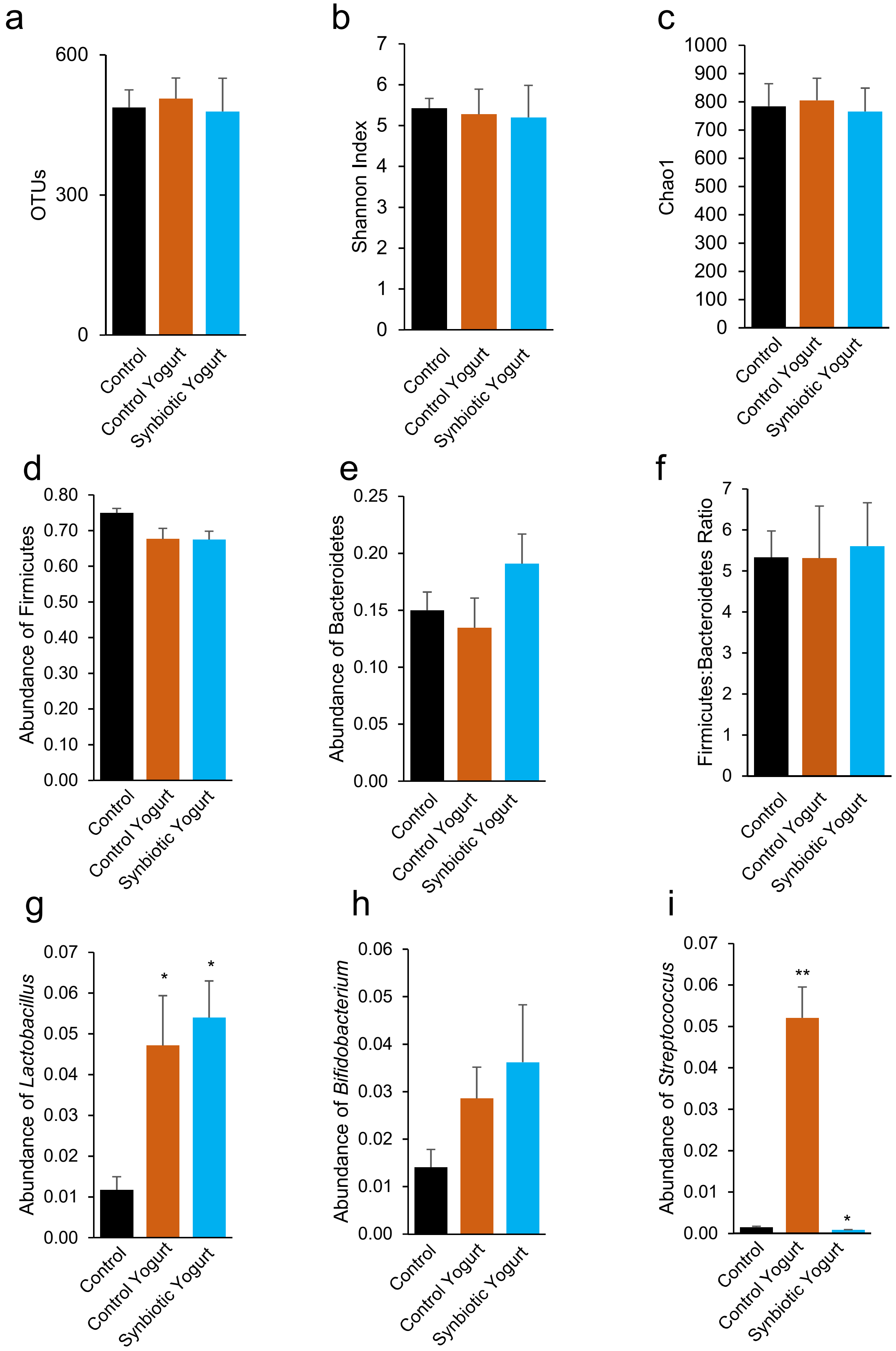

Supplementary Figure S2. Control and synbiotic yogurt feeding does not significantly affect α-diversity measures but modulates the gut microbiome at the phyla and species levels. (a-c) Measures of α-diversity including operational taxonomic units (OTUs) (a), Shannon index (b), and Chao1 (c). (d,e) Abundances of Firmicutes (d) and Bacteroidetes (e), two major bacterial phyla. (f) Firmicutes:Bacteroidetes ratio. (g,h) Abundances of probiotic bacteria *Lactobacillus* (g) and *Bifidobacterium* (h). (i) Relative abundance of *Streptococcus*. Values are mean ± standard error of the mean (error bars). Values with *P<0.05; **P<0.001 are statistically significant.
